# Clinical Outcomes of Dupilumab-Associated Ocular Surface Disease in the Paediatric Population

**DOI:** 10.7759/cureus.97878

**Published:** 2025-11-26

**Authors:** Vivien Nguyen, Susan Zhang, Shuan Dai

**Affiliations:** 1 Ophthalmology, Queensland Children's Hospital, Brisbane, AUS; 2 Ophthalmology, Sydney and Sydney Eye Hospital, Sydney, AUS

**Keywords:** atopic dermatitis, atopy, conjunctivitis, dupilumab-associated ocular surface disease (daosd), eczema, paediatric atopic dermatitis

## Abstract

Background

The aim of this study is to characterise dupilumab-associated ocular surface disease (DAOSD) and its management in the paediatric population.

Methods

Retrospective data were obtained from the electronic medical records of a tertiary Australian paediatric public hospital’s Ophthalmology department for patients reviewed between 1 January 2021 and 1 December 2024. Demographic information was recorded, including gender, age when dupilumab was started, ethnicity, dupilumab indication, past medical history, and baseline serology testing. Clinical characteristics collected included presenting visual acuity (VA), ophthalmological findings, ocular treatment, time to onset and resolution of DAOSD, and final ophthalmological outcome. Cessation of dupilumab due to DAOSD was also noted.

Results

A total of 13 patients were identified with DAOSD. The mean time from starting dupilumab to onset of DAOSD was 24 weeks (7.3-97.9 weeks). The mean time from onset of ocular symptoms to ophthalmology review was 3.6 weeks (range 0.1-8). The mean baseline VA was logMAR 0.01 (range 0-0.3). Ophthalmological examination findings included conjunctival injection (n=9, 69%), papillary reaction (n=5, 39%), superficial punctate keratitis (n=5, 39%), limbitis (n=3, 23%), and periocular involvement (n=2, 14%). Management included lubricant eye drops (n=13, 100%), topical steroid drops (n=9, 80%), topical antihistamines/mast cell stabiliser eye drops (n=5, 39%), and systemic azithromycin (n=1, 8%). The mean final VA was logMAR 0.06 (range 0-0.3). Three (23%) patients discontinued dupilumab due to ocular or systemic side effects.

Conclusions

Early ophthalmological assessment and intervention can lead to positive final visual outcomes in paediatric patients experiencing DAOSD. Future studies should investigate the long-term effects of DAOSD treatment in the paediatric population.

## Introduction

Dupilumab is a monoclonal antibody with increasing use in type 2 inflammatory diseases such as atopic dermatitis, eosinophilic oesophagitis, and chronic rhinosinusitis [1]. Dupilumab-associated ocular surface disease (DAOSD) has been reported in both the adult and paediatric populations, with potentially significant impacts on quality of life that may lead to therapy discontinuation [2-7]. DAOSD is a collective term used to describe ocular surface disease arising from the use of dupilumab, such as conjunctivitis, blepharitis, and keratitis, with conjunctivitis being the most common manifestation [2,6,8]. There has been varying incidence of DAOSD reported in the literature, from 8.6% to 22.1% in clinical trials and 0.5% to 70% in real-world data [9]. Some studies report a higher incidence of DAOSD in dupilumab-treated groups compared with placebo groups (22.1% vs 11.1%), whereas others did not observe this difference [3]. Reported risk factors for DAOSD include more severe baseline atopic disease, previous history of conjunctivitis, facial or eyelid eczema, and elevated baseline serum biomarkers (thymus and activation-regulated chemokine [TARC], eosinophils, and immunoglobulin E [IgE]) [3,6,10]. Co-management with ophthalmology is therefore essential to reduce the risk of significant ocular complications such as cicatrisation, punctal stenosis, and corneal scarring [2]. Despite the growing body of literature, current paediatric research is limited by inconsistent ocular assessment and a lack of reported visual outcomes, with ocular findings often not being the primary or pre-specified outcome [7,8,11]. There is also a lack of standardised ophthalmic evaluation by ophthalmology practitioners and limited data on the impacts and visual outcomes for the paediatric population. Given the increasing utilisation of dupilumab in clinical practice for children with type 2 inflammatory disease, an anticipated rise in DAOSD is also expected. The aim of this study is to help characterise DAOSD and its management in the paediatric population, alongside analysing visual outcomes as a primary measure.

## Materials and methods

Retrospective data were obtained from patients who were on dupilumab and reviewed by the ophthalmology department at the Queensland Children’s Hospital, a tertiary paediatric public hospital, between 1 January 2021 and 1 December 2024. Inclusion criteria included participants aged <18 years at their first presentation to the ophthalmology department and had DAOSD confirmed by ophthalmology. Exclusion criteria included participants who were >18 years old without clinical evidence or ophthalmology confirmed DAOSD. After the criteria were applied, there were 13 patients (26 eyes) remaining in the study. The demographic information, clinical characteristics and visual outcomes were collected via the electronic medical record (EMR). Ethics approval for the study was granted by the Institutional Human Research Ethics Committee (HREC/2025/QCHQ/118828). This study adhered to the principles outlined in the Declaration of Helsinki.

Baseline demographic information, including gender, age when dupilumab was started, ethnicity, dupilumab indication, past medical history, and baseline serology testing (eosinophil count and IgE levels), were obtained from the EMR. Clinical characteristics collected included presenting visual acuity (VA), ophthalmological findings, ocular treatment, onset and resolution of DAOSD, and final ophthalmological outcome. Resolution of DAOSD was defined as the period from initiation of ophthalmology-directed treatment to the point at which the ocular condition was deemed quiescent by the ophthalmology practitioner. Final VA was defined as the VA when the resolution of DAOSD was determined. Cessation of dupilumab due to DAOSD was also noted. Dupilumab dosages and frequencies varied and were determined by the paediatric dermatology department. This was often an initial loading dose (400-600 mg) followed by a treatment dose (200-300 mg) on a fortnightly or monthly basis. The collected data were analysed using SPSS Statistics Version 27 (IBM Corp., Armonk, NY). Descriptive statistics was generated for population demographics.

## Results

Patient baseline characteristics are presented in Table 1. A total of 13 patients (26 eyes) were identified with DAOSD through the paediatric ophthalmology department and received a full ophthalmic examination. The average age at which dupilumab was initiated was 9.1 years (range 1.7-15.7 years), with most patients being male (85%) and Caucasian (85%). The majority had been referred in by the dermatology department (85%) and had been started on dupilumab for atopic dermatitis (92%). Some patients did not have a previous ocular history; however, 23% had previously documented vernal keratoconjunctivitis (VKC). Almost half of the patients had asthma, and 77% of patients had a known allergy to food, environmental trigger and/or medication. The Eczema Area and Severity Index (EASI) score is a standardised evaluation tool for the severity of atopic dermatitis signs [12]. The scoring is as follows: no disease (score=0), mild disease (score=1.1-7), moderate disease (score=7.1-21), severe disease (score=21.1-50), and very severe disease (score >51). The mean EASI score for participants in this study was 37.14. All patients had marked eosinophilia and significantly elevated IgE levels.

**Table 1:** Baseline demographic characteristics of paediatric patients with DAOSD. ^a^Eight (61.5%) patients had more than three allergies. ^b^Nine (69.2%) patients had more than two systemic steroids DAOSD, dupilumab-associated ocular surface disease; EASI, Eczema Area and Severity Index; IgE, immunoglobulin E

Characteristic		Value
Age in years: mean (range)		9.1 (1.7–15.7)
Gender, n (%)	Male	11 (84.6)
	Female	2 (15.4)
Ethnicity, n (%)	Caucasian	11 (84.6)
	Asian	2 (15.4)
Dupilumab indication, n (%)	Atopic dermatitis (severe)	12 (92.3)
	Asthma (severe)	1 (17.7)
Referral source, n (%)	Dermatology	11 (84.6)
	Emergency department	1 (7.7)
	General practitioner	1 (7.7)
Atopy/allergy, n (%)^a^	Asthma	6 (46.2)
	Food allergy	9 (69.2)
	Environmental allergy	5 (38.5)
	Drug allergy	1 (7.7)
Systemic steroids, n (%)^b^	Any	11 (84.6)
	Betamethasone dipropionate 0.05%	9 (69.2)
	Methylprednisolone aceponate 0.1%	7 (53.8)
	Hydrocortisone 1%	1 (7.7)
Facial dermatitis, n (%)		12 (92.3)
Vernal keratoconjunctivitis, n (%)		3 (23.1)
EASI score (n=12), mean (range)		37.14 (10.2–61.2)
Eosinophil x10^9^/L (n=12), mean (range)		1.73 (0.50–3.33)
IgE kU/L (n=12), mean (range)		5,356.64 (456–10,300)

Ophthalmic characteristics are presented in Table 2. The mean time from starting dupilumab to onset of DAOSD was 24 weeks (7.3-97.9 weeks). The mean time from onset of ocular symptoms to ophthalmology review was 3.6 weeks (range: 0.1-8). The mean baseline VA was logMAR 0.09 (range: 0-0.3). The most frequently reported ocular symptoms were ocular pain/discomfort (69%) and pruritis (31%). Ophthalmological examination findings included conjunctival injection (n=9, 69%), papillary reaction (n=5, 39%), superficial punctate keratitis (n=5, 39%), limbitis (n=3, 23%), and periocular involvement (n=2, 14%). Ophthalmology-initiated treatment consisted of a combination of topical antihistamines/mast cell stabilisers, topical steroids, preservative-free lubricants, and oral antibiotics with variable frequencies. All 13 (100%) patients were prescribed preservative-free lubricant eye drops, nine (69%) patients with topical steroid eye drops, five (39%) patients with topical antihistamines or mast cell stabiliser eye drops, and one (8%) patient with systemic azithromycin. Preservative-free lubricant eye drops included carmellose sodium 0.5%, sodium hyaluronate 0.2%, and sodium cromoglycate 2%. Topical steroids prescribed included fluorometholone 1% eye drops (62%), dexamethasone 0.1% eye drops (8%), or hydrocortisone 1% ointment (8%), ranging from daily to four times a day. The frequency of topical steroid administration was transitioned for some patients or was switched to a different topical steroid pending on clinical progress, with an aim to wean off all drops where possible. The mean final VA was logMAR 0.06 (range: 0-0.3). The mean time to DAOSD resolution was 9.9 weeks (range: 2.7-20.8).

**Table 2:** Ophthalmic characteristics of paediatric patients with DAOSD. ^a^Ten (76.9%) patients had two or more ocular symptoms; ^b^Nine (69.2%) patients had two or more ocular findings. DAOSD, dupilumab-associated ocular surface disease

Characteristic		Value
Duration from dupilumab initiation to DAOSD (weeks), mean (range)		24 (7.3–97.9)
Duration from DAOSD to ophthalmic review (weeks), mean (range)		3.6 (0.1–8.0)
Ocular symptoms, n (%)^a^	Pain/discomfort	9 (69.2)
	Pruritis	4 (30.8)
	Dryness	3 (23.1)
	Photosensitivity	2 (14.4)
Ocular findings, n (%)^b^	Conjunctival injection	9 (69.2)
	Papillary reaction	5 (38.5)
	Superficial punctate keratitis	5 (38.5)
	Limbitis	3 (23.1)
	Periocular swelling	2 (14.4)
Visual acuity at first visit (logMAR), mean (range)		0.09 (0–0.3)
Ocular treatment, n (%)	Lubricating eye drops	13 (100.0)
	Corticosteroid eye drops/ointment (e.g., fluorometholone 1%, dexamethasone 0.1%, hydrocortisone 1%)	9 (69.2)
	Antihistamine/mast cell stabiliser eye drops (e.g., olopatadine 0.1%, ketotifen 1.0%)	5 (38.5)
	Oral antibiotics	1 (7.7)
Visual acuity at resolution visit (logMAR), mean (range)		0.06 (0–0.3)
Duration to DAOSD stability/resolution (weeks), mean (range)		9.9 (2.7–20.8)

Three patients had to discontinue dupilumab for various reasons. One patient ceased due to experiencing a recurrent shield ulcer on the background of previous VKC prior to starting dupilumab. The second patient ceased due to no systemic improvement in their atopic dermatitis; this patient is currently awaiting genetics review to exclude a genetic cause for their resistant atopic disease. The third patient ceased dupilumab due to experiencing recurrent chest pain and severe anaphylactic-like reactions on a background of extensive food, environmental, and drug allergies.

## Discussion

Our study reports that early ophthalmic intervention is critical in reducing the severity of DAOSD and preventing associated sequelae. The ocular findings and treatments prescribed for DAOSD in this cohort were also consistent with those reported in adult literature. Recent paediatric cohort studies from the Netherlands and Singapore examined all dupilumab-treated patients rather than specifically those with DAOSD. Awareness of DAOSD is particularly important in paediatric patients, as challenges in symptom reporting may delay diagnosis. Optometric or ophthalmological assessment prior to dupilumab initiation is essential to optimise baseline ocular surface health and identify high-risk patients.

A 2019 analysis of six randomised placebo-controlled clinical trials revealed that patients with atopic dermatitis treated with dupilumab had a higher incidence of DAOSD compared to other conditions such as asthma, chronic rhinosinusitis, and eosinophilic oesophagitis [3]. This was reflected in this reported study, with the majority of patients having atopic dermatitis. In previous studies, symptoms typically occurred 14 days after the initiation of dupilumab monotherapy and between 4 and 8 weeks after the initiation of combination management with systemic corticosteroids [3]. A 2025 prospective paediatric study reported the onset of associated ocular symptoms at 13 weeks, with 86% of patients developing DAOSD within the first 52 weeks of dupilumab initiation [7]. In the reported study, the onset of DAOSD occurred later than current literature, with a median of 24 weeks (7-98 weeks) after initiation of dupilumab; however, 85% of patients were also receiving concurrent corticosteroids, which may have delayed symptom onset. Referral to the ophthalmology department within three months from initiation of dupilumab therapy may be beneficial given the potentially challenging nature of ocular symptom self-reporting in paediatric patients.

In the reported study, the mean EASI score was 37 (severe disease), with 23% (n = 3) of patients classified with very severe disease. This is consistent with literature reporting that higher EASI scores are associated with an increased risk of DAOSD development [12,13]. In the reported study, the mean baseline IgE level was 5,357 kU/L, and all atopic dermatitis patients reviewed by the ophthalmology department had severe disease. The mean eosinophil count was 1.73 × 10⁹/L, which is also markedly elevated compared with the normal range of 0.02-0.5 × 10⁹/L. The presence of periocular dermatitis has been described to have an association with more severe forms of DAOSD in adults [13,14]. In our study, 92% of DAOSD patients had facial dermatitis at initial ophthalmology review. The only patient without facial dermatitis had commenced dupilumab for severe asthma rather than atopic dermatitis. Although DAOSD is rarely reported in patients without atopic dermatitis, this finding demonstrates that it can still occur in dupilumab-treated patients for other indications.

DAOSD ocular signs and symptoms appear to be similar in adult and paediatric populations [8,15]. In the reported study, the most common ocular symptoms were pain or discomfort (69%) and pruritus (31%), consistent with findings in the current literature. A 2025 prospective study of paediatric atopic dermatitis patients treated with dupilumab found that the most common ocular symptoms were pruritus (75%), redness (72%), and tearing (58%) [7]. Similarly, a 2025 retrospective review of Asian children and adolescents on dupilumab comparably reported high incidences of ocular redness (87.5%) and pruritus (43.8%) [2]. With regard to ocular signs, the most commonly reported in literature include conjunctival injection (62%), superficial punctate keratitis (55%), and papillary reaction (28%) [5]. This was consistent with our findings, where conjunctival injection (69%), papillary reaction (39%), and superficial punctate keratitis (39%) were the most common signs observed. Rarer sequelae such as punctal stenosis and severe conjunctival cicatrisation have been reported in past literature [14,16]. These cases, however, were all described in adult patients and had reported DAOSD symptoms persisting for more than five weeks (range: 4-104 weeks) prior to seeking ophthalmological intervention [14]. By contrast, in the reported study, the mean time from DAOSD symptom onset to ophthalmological review was shorter at 3.6 weeks (range: 0.1-8.0 weeks), with no cases of punctal stenosis or conjunctival cicatrisation observed. Early detection and management of DAOSD are therefore paramount in reducing the inflammatory ocular surface processes that predispose cicatricial changes.

**Figure 1:**
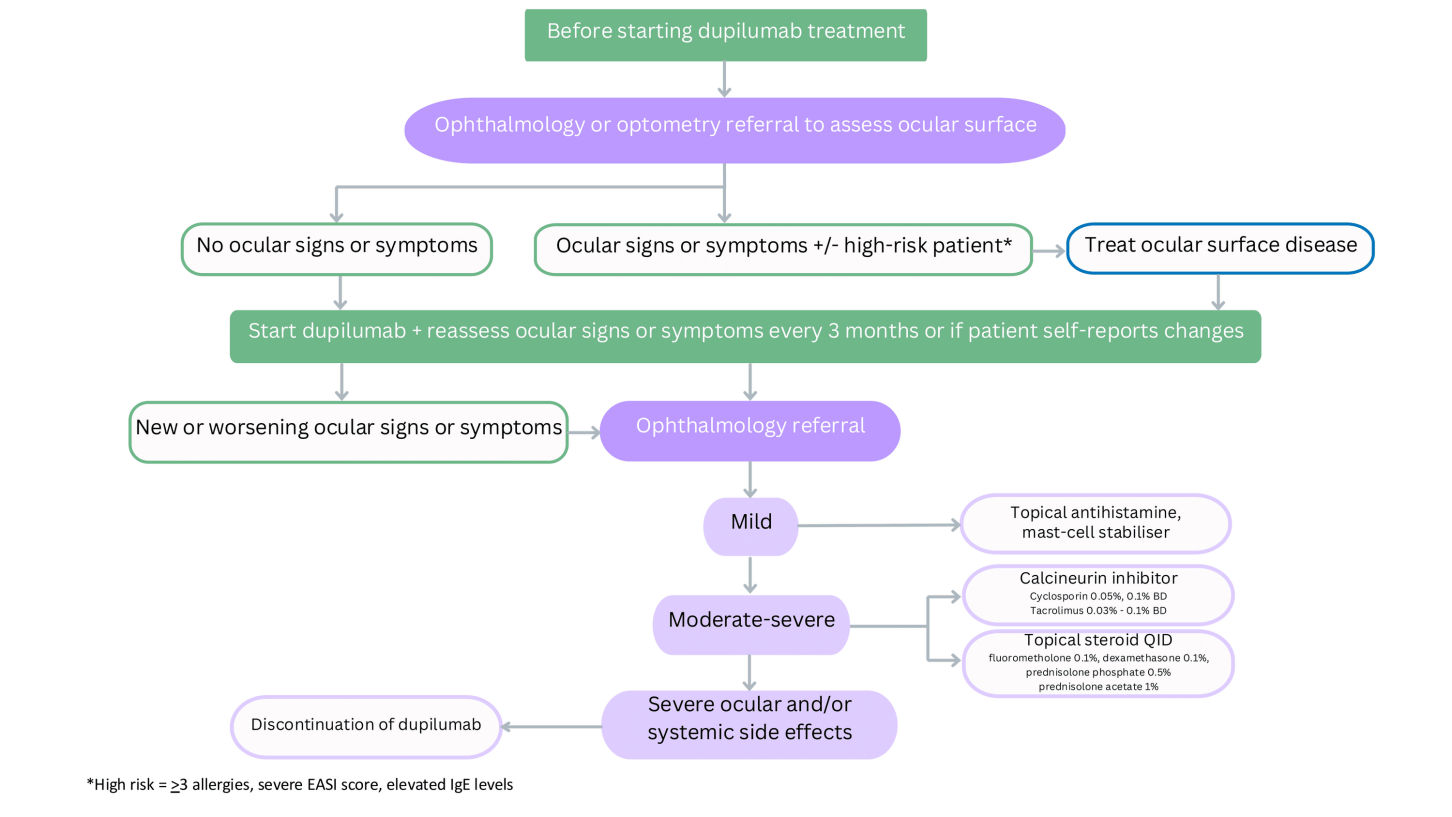
Suggested screening pathway of paediatric patients starting dupilumab treatment and the management of DAOSD. Note: Figure created by the authors DAOSD, dupilumab-associated ocular surface disease

Management of DAOSD in the reported study varied amongst patients and included topical antihistamine/mast cell stabiliser eye drops, topical steroid eye drops, topical lubricant eye drops, and systemic antibiotics. The intensity and combination of treatment were clinician-dependent and based on the severity of ocular disease. In 2023, Rampersad et al. conducted a retrospective review of 46 older paediatric patients (9-14 years old), with 12 patients developing DAOSD [17]. Of these 12 patients, topical treatment included ciclosporin, steroid, lubrication, and chloramphenicol [17]. Variation in management could be reduced by developing institutional protocols and guidelines, such as those previously suggested by Foley et al. [9]. We propose our own screening protocol tailored for the paediatric population in Figure 1. Involvement of community eye health professionals in screening for ophthalmological signs is particularly important in the paediatric population, where patients may not be able to self-report symptoms, or where access to ophthalmology care may be delayed or restricted.

In the reported study, 77% (n = 10) of patients demonstrated an excellent response to ophthalmology-led management of DAOSD. The remaining 23% (n = 3) of patients required cessation of dupilumab for ocular (n = 1) or systemic reasons (n = 2). Contrastingly, literature reports very minimal cessation rates of dupilumab due to ocular side effects [16,18,19]. In the reported study, two patients needed to cease dupilumab due to systemic reasons: one developed severe anaphylaxis with chest pain, and the other showed no systemic improvement and was transitioned to methotrexate. The single patient who discontinued due to ocular side effects had developed a recurrent shield ulcer on a background of known VKC, necessitating cessation of dupilumab. Patients with pre-existing VKC or blepharokeratoconjunctivitis may therefore be at a higher risk of severe DAOSD, and dupilumab use in this cohort should be approached with caution. Notably, this was also the only patient in this study who was prescribed dupilumab for severe asthma. Future research should investigate the incidence of paediatric DAOSD and its potential association with dupilumab use for conditions beyond atopic dermatitis.

The visual prognosis of DAOSD patients is reported to be excellent [16]. This was similar in the reported study, with initial mean baseline VA and final VA at logMAR 0.09 and 0.06, respectively. Most patients in this report tolerated ocular treatment for DAOSD, with only one patient developing a steroid response and subsequently transitioning to ciclosporin eye drops. A 2021 retrospective analysis of atopic dermatitis patients treated with dupilumab via the dermatology department reported no ocular side effects from steroid eye drops [16]. Given that the majority of DAOSD studies in the current literature are driven by dermatologists, it is unclear what the true incidence of ocular steroid responses is in DAOSD-treated patients, and future studies should analyse other confounding factors. Younger patients with juvenile idiopathic arthritis-associated uveitis are particularly susceptible to topical steroid-related complications, including cataract and elevated intraocular pressure [20]. Evidence on the long-term outcomes of these sequelae in paediatric patients receiving DAOSD management, which frequently requires topical steroids, is however limited. Dupilumab prescribers should therefore routinely discuss the potential ocular side effects and their management with patients before initiating treatment.

This study was limited by being retrospective in nature. As this study was conducted at a tertiary paediatric ophthalmology service, the severity of DAOSD in this cohort is likely higher than in the general population, as these children may have had more complex atopic disease. Selection bias was also present, as ophthalmology referrals were only made for patients who developed ocular signs or symptoms identified by another healthcare provider. Future studies should include larger cohorts with longer follow-up to improve prognostication and risk stratification of paediatric DAOSD. Collaborative research with respiratory and dermatology services would facilitate better screening for DAOSD in all dupilumab-treated patients, enabling earlier intervention and more comprehensive data collection.

## Conclusions

Early ophthalmological assessment and intervention can lead to positive final VA outcomes in paediatric patients experiencing DAOSD. With the increasing use of dupilumab in the paediatric population, DAOSD awareness within respiratory and dermatology services may help enable earlier intervention. Collaboration with other eye care professionals, such as community optometry, is also paramount. Future studies should investigate the long-term effects of treatment, such as the ocular steroid response, for DAOSD in the paediatric population.
